# Supplementary material for: PPP3CB overexpression mediates EGFR TKI resistance in lung tumors via calcineurin/MEK/ERK signaling
Source: Life Sci Alliance. 2024 Oct 1;7(12):e202402873. doi: 10.26508/lsa.202402873 (PMC11447527; doi:10.26508/lsa.202402873)
Supplement: Supplementary file 1 [file LSA-2024-02873_SdataFS1.pptx]

## Slide 1
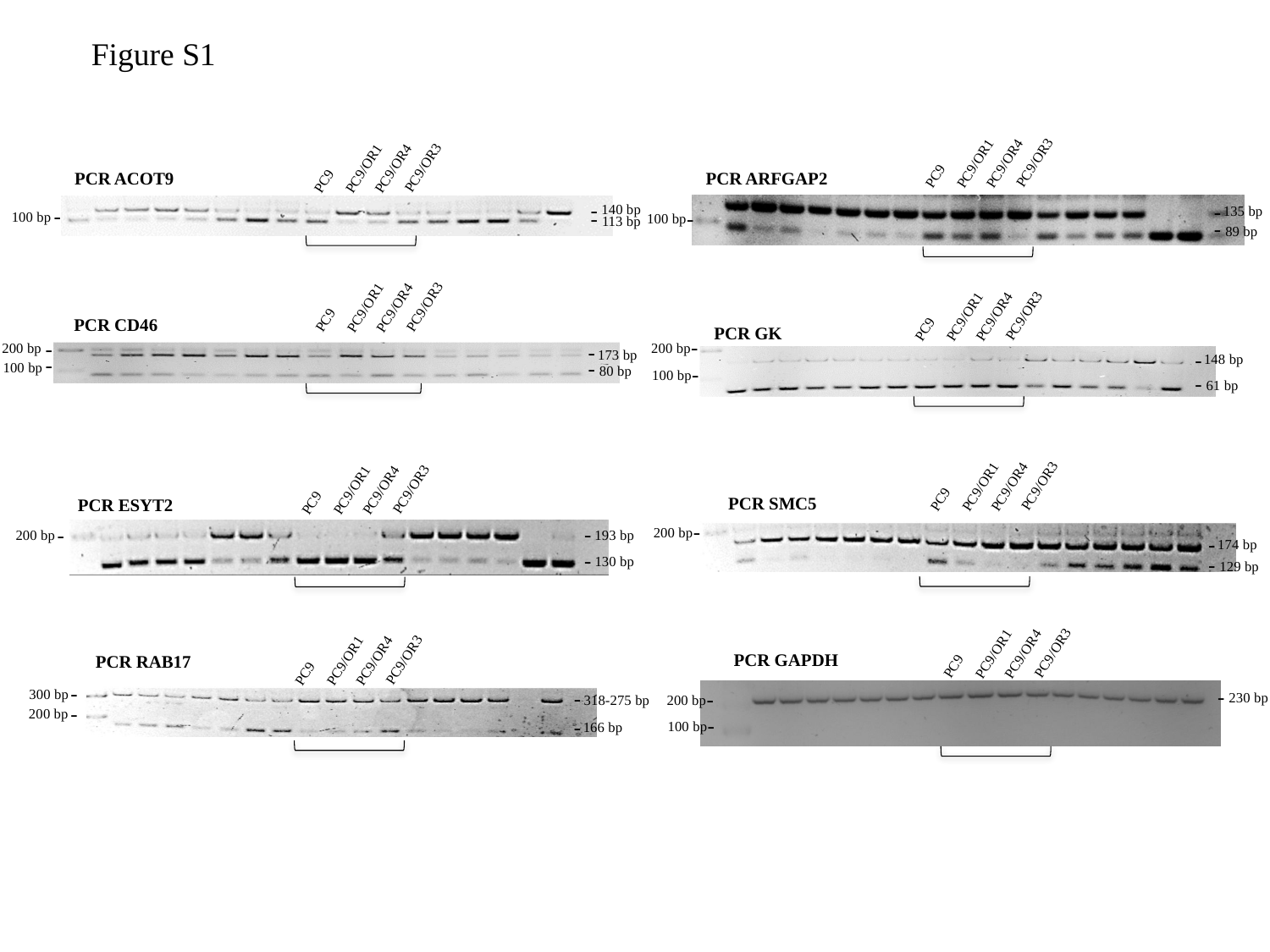

Figure S1
PC9/OR3
PC9/OR1
PC9/OR4
PC9
PC9/OR3
PC9/OR1
PC9/OR4
PC9
PCR ACOT9
PCR ARFGAP2
-
-
140 bp
135 bp
-
-
-
100 bp
100 bp
113 bp
-
89 bp
PC9/OR3
PC9/OR1
PC9/OR4
PC9
PC9/OR3
PC9/OR1
PC9/OR4
PC9
PCR CD46
PCR GK
-
-
-
200 bp
200 bp
173 bp
-
148 bp
-
-
100 bp
-
80 bp
100 bp
-
61 bp
PC9/OR3
PC9/OR1
PC9/OR4
PC9
PC9/OR3
PC9/OR1
PC9/OR4
PC9
PCR SMC5
PCR ESYT2
-
-
-
200 bp
193 bp
200 bp
-
174 bp
-
-
130 bp
129 bp
PC9/OR3
PC9/OR1
PC9/OR4
PC9
PC9/OR3
PC9/OR1
PC9/OR4
PC9
PCR GAPDH
PCR RAB17
-
-
300 bp
-
-
230 bp
200 bp
318-275 bp
-
200 bp
-
-
100 bp
166 bp
