# Supplementary material for: PPP3CB overexpression mediates EGFR TKI resistance in lung tumors via calcineurin/MEK/ERK signaling
Source: Life Sci Alliance. 2024 Oct 1;7(12):e202402873. doi: 10.26508/lsa.202402873 (PMC11447527; doi:10.26508/lsa.202402873)
Supplement: Supplementary file 2 [file LSA-2024-02873_SdataF1.pdf]

Figure 1 A

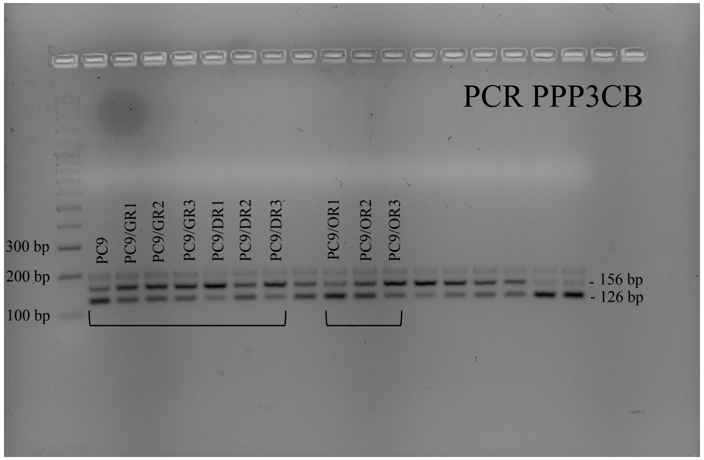

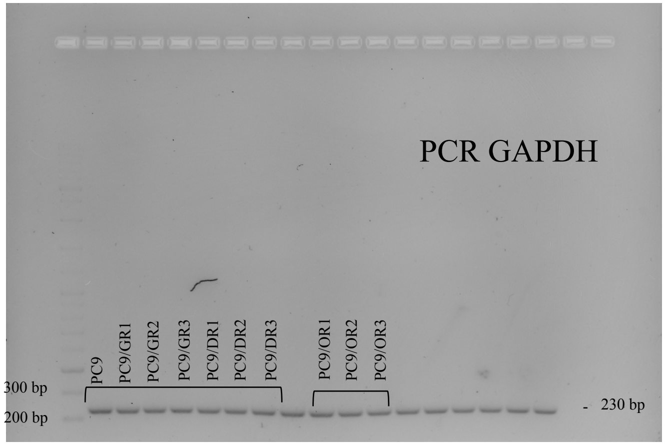

	PC9	PC9/ GR1	PC9/ GR2	PC9/ GR3	PC9/ DR1	PC9/ DR2	PC9/ DR3	PC9/ OR1	PC9/ OR2	PC9/ OR3
PSI	0.29	0.61	0.63	0.68	0.85	0.51	0.8	0.19	0.46	0.73
	0.17	0.47	0.38	0.37	0.86	0.58	0.5	0.21	0.21	0.44
	0.17	0.31	0.24	0.25	0.53	0.32	0.5	0.11	0.3	0.5

Figure 1 B

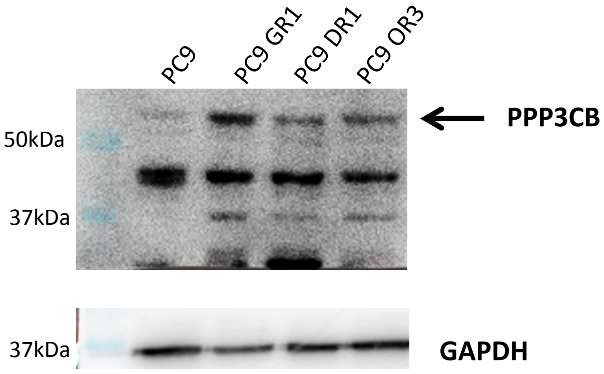

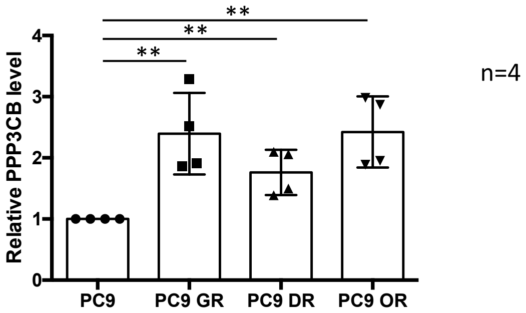

Figure 1 C

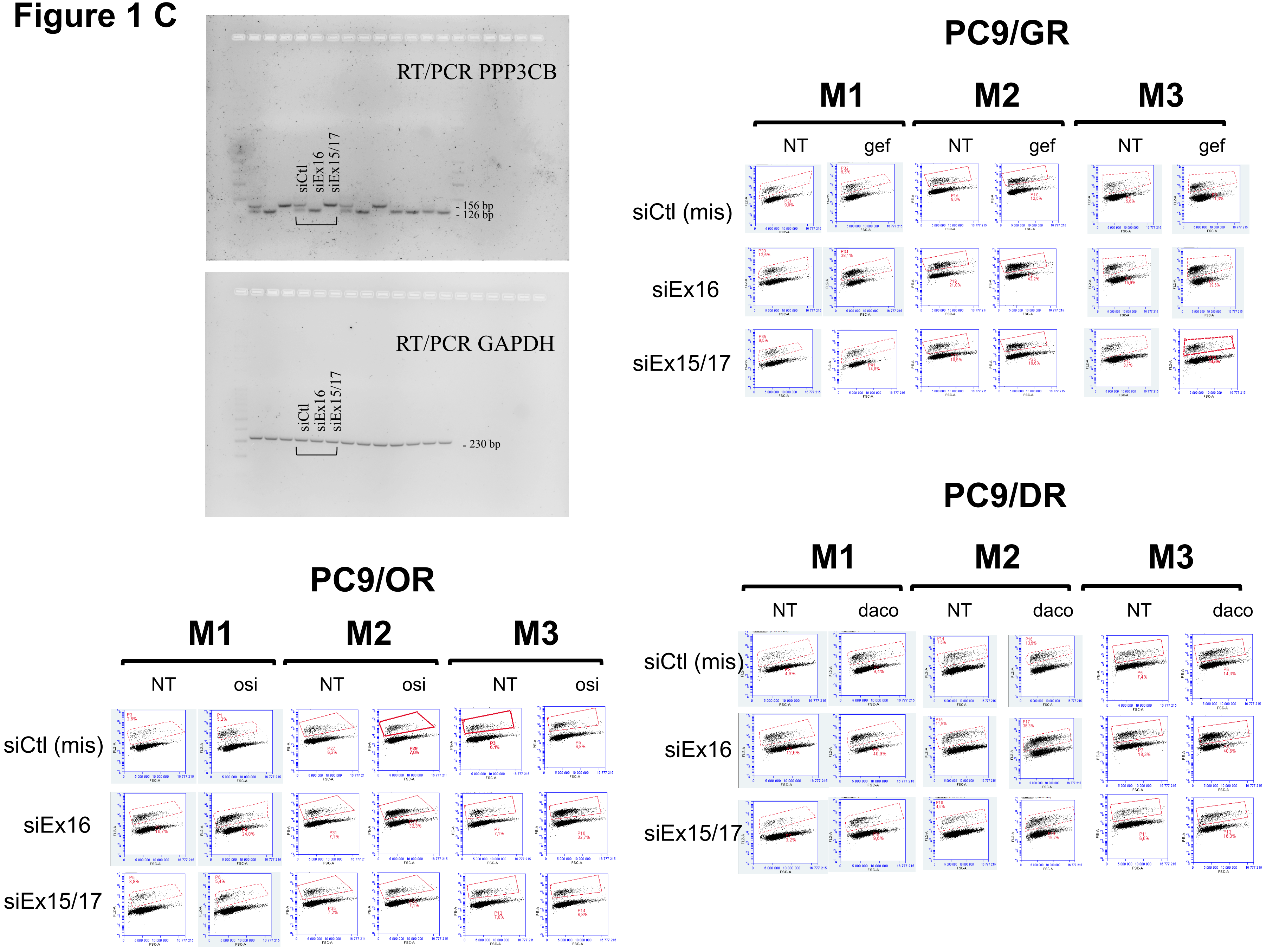
RT/PCR PPP3CB
siCtl
siEx16
siEx15/17
156 bp
126 bp
RT/PCR GAPDH
siCtl
siEx16
siEx15/17
230 bp
PC9/GR
M1
M2
M3
NT
gef
NT
gef
NT
gef
siCtl (mis)
siEx16
siEx15/17
PC9/DR
M1
M2
M3
NT
daco
NT
daco
NT
daco
siCtl (mis)
siEx16
siEx15/17
PC9/OR
M1
M2
M3
NT
osi
NT
osi
NT
osi
siCtl (mis)
siEx16
siEx15/17

Figure 1 D

PC9/GR

	siCtl NT	siCtl gef	siEx16 NT	siEx16 gef
M1	100	98	102	48
M2	100	95	100	45,5
M3	100	101	105	41,7

PC9/DR

	siCtl NT	siCtl daco	siEx16 NT	siEx16 daco
M1	100	93	100	45
M2	100	89	97	49
M3	100	108	99	53,8

PC9/OR

	siCtl NT	siCtl osi	siEx16 NT	siEx16 osi
M1	100	97	96	47
M2	100	97	107	51
M3	100	108	111,4	52,6

Figure 1 E

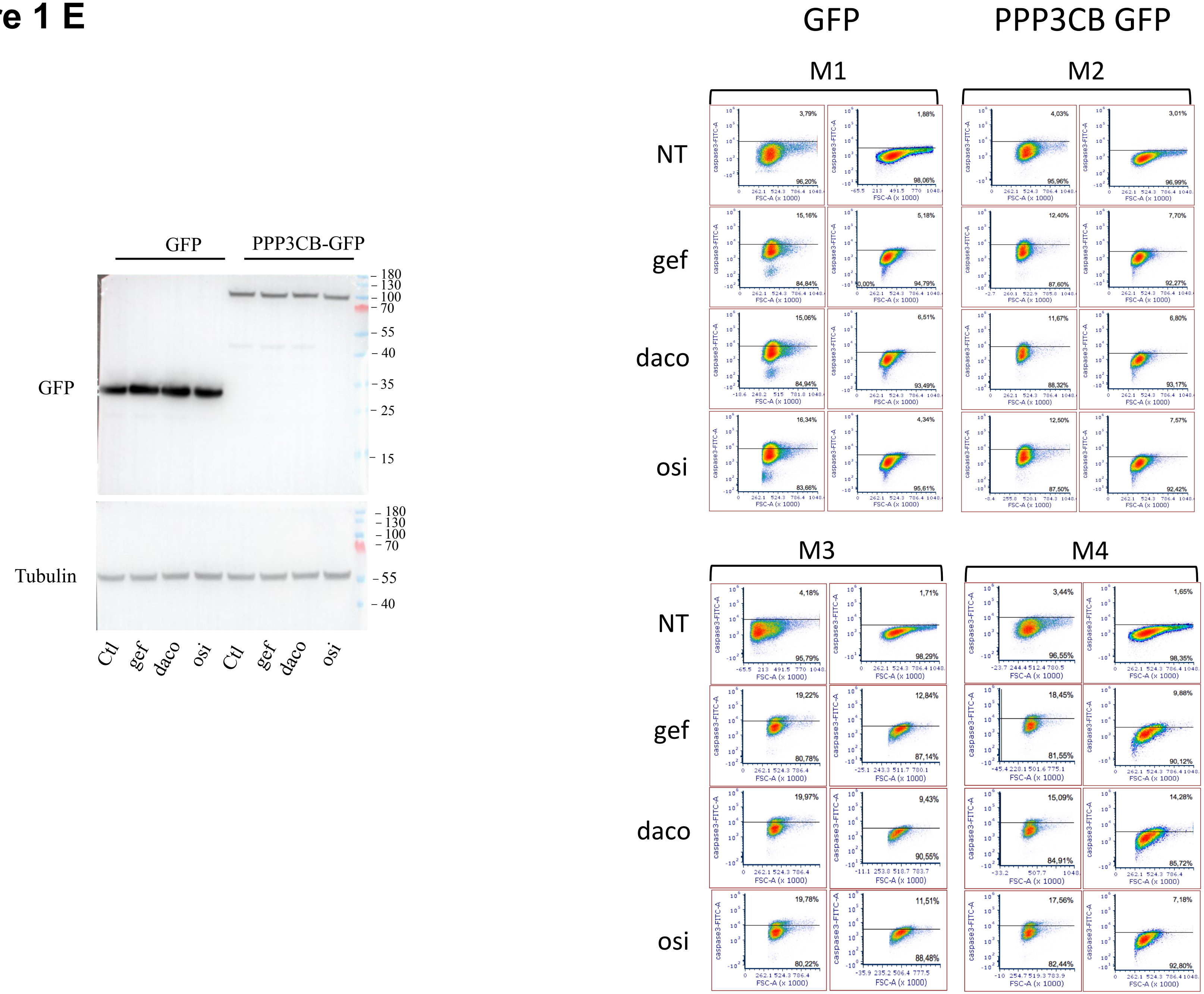
GFP
PPP3CB-GFP
180
130
100
70
55
40
35
25
15
GFP
Tubulin
Ctl
gef
daco
osi
Ctl
gef
daco
osi
GFP
PPP3CB GFP
M1
M2
M3
M4
NT
gef
daco
osi
NT
gef
daco
osi
caspase3-FITC-A
FSC-A (x 1000)
3,79%
96,20%
1,88%
98,06%
4,03%
95,96%
3,01%
96,99%
15,16%
84,84%
5,18%
0,00%
94,79%
12,40%
87,60%
7,70%
92,27%
15,06%
84,94%
6,51%
93,49%
11,67%
88,32%
6,80%
93,17%
16,34%
83,66%
4,34%
95,61%
12,50%
87,50%
7,57%
92,42%
4,18%
95,79%
1,71%
98,29%
3,44%
96,55%
1,65%
98,35%
19,22%
80,78%
12,84%
87,14%
18,45%
81,55%
9,88%
90,12%
19,97%
9,43%
90,55%
15,09%
84,91%
14,28%
85,72%
19,78%
80,22%
11,51%
88,48%
17,56%
82,44%
7,18%
92,80%
