# Supplementary material for: PPP3CB overexpression mediates EGFR TKI resistance in lung tumors via calcineurin/MEK/ERK signaling
Source: Life Sci Alliance. 2024 Oct 1;7(12):e202402873. doi: 10.26508/lsa.202402873 (PMC11447527; doi:10.26508/lsa.202402873)
Supplement: Supplementary file 3 [file LSA-2024-02873_SdataF2.pdf]

Figure 2 B

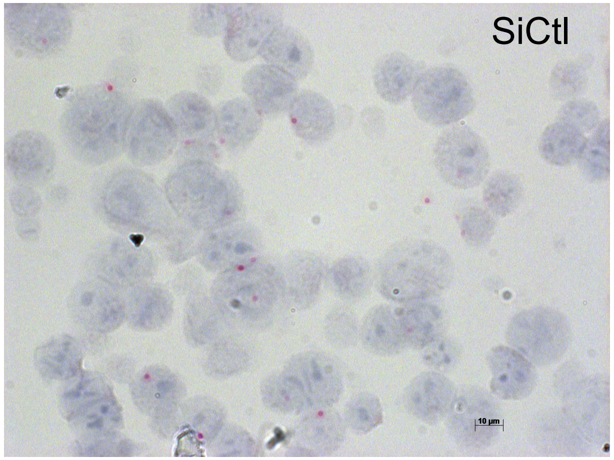

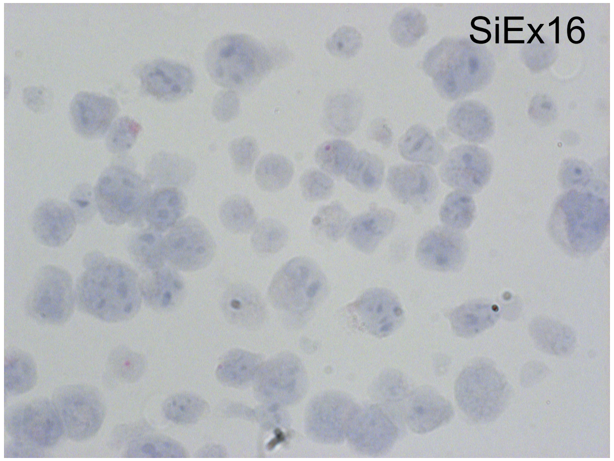

Figure 2 C

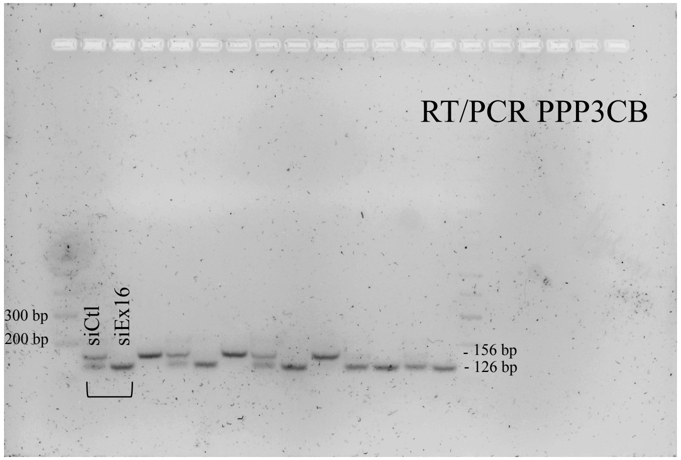

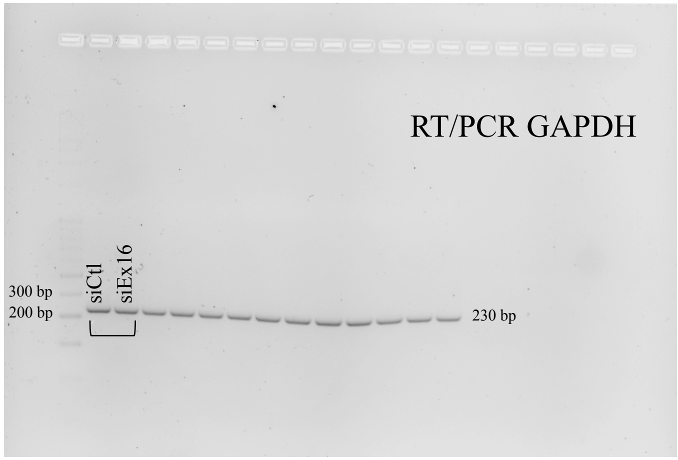

% of cells with dots

PC9 OR siCtl	PC9 OR siex16
36.36	5.35
21.27	11.53
52.94	3.63
35	2.7
11.76	4.87
30.76	6.38
20.51	3.03
39.53	0
13.55	0
51.42	0
43.75	6.81

Figure 2 D

Pre-treatment

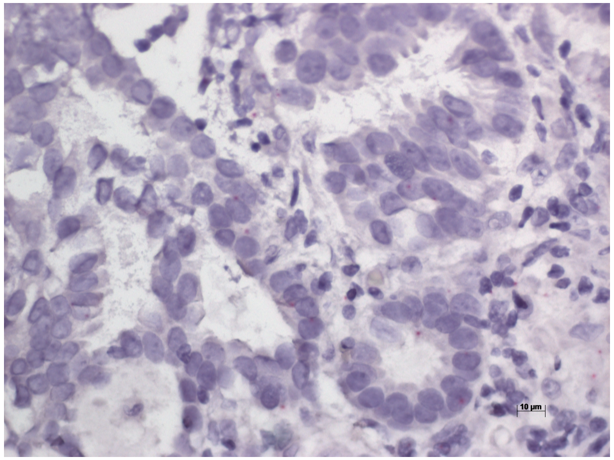

Post-treatment

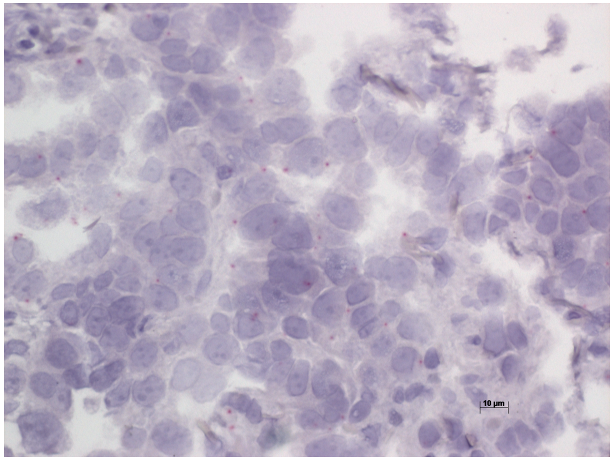

Figure 2 E

positive samples

% cells with dots

baseline	PD
1,94	7,89
0,23	5,04
3,27	8,3
6,72	20,97
0,77	4,62
0,41	26,61
11,47	20,24
0,59	7,45
0	1,82
0,77	4,62
4,9	21,25
