# Supplementary material for: PPP3CB overexpression mediates EGFR TKI resistance in lung tumors via calcineurin/MEK/ERK signaling
Source: Life Sci Alliance. 2024 Oct 1;7(12):e202402873. doi: 10.26508/lsa.202402873 (PMC11447527; doi:10.26508/lsa.202402873)
Supplement: Supplementary file 4 [file LSA-2024-02873_TableS1.pdf]

Supplemental Table 1. PPP3CB status in 25 paired biopsies subcohort of patients treated with first-line EGFR TKI

	PPP3CB status (% of cells with dots)	
Patients	Baseline	post-EGFR TKI
1	7.58	3.43
2	1.08	2.45
3	4.9	21.25
4	1.45	1.5
5	0.23	5.04
6	2.13	2.25
7	0.86	0.28
8	9.04	5.33
9	5.34	2.64
10	2.99	3.9
11	0.77	4.62
12	1.94	7.89
13	0	1.82
14	6.72	20.97
15	3.27	8.3
16	1.08	3.92
17	0.77	4.62
18	5.22	0.55
19	2.1	1.87
20	3.05	0.93
21	0.35	0
22	22.54	2.33
23	0.41	26.61
24	0.59	7.45
25	1.37	0.2
