# Supplementary material for: PPP3CB overexpression mediates EGFR TKI resistance in lung tumors via calcineurin/MEK/ERK signaling
Source: Life Sci Alliance. 2024 Oct 1;7(12):e202402873. doi: 10.26508/lsa.202402873 (PMC11447527; doi:10.26508/lsa.202402873)
Supplement: Supplementary file 5 [file LSA-2024-02873_SdataF3.pdf]

# Figure 3 A

**PC9/GR**

| gef (μM) | DMSO | | | | CsA 1μM | | | | CsA 5 μM | | | |
|---|---|---|---|---|---|---|---|---|---|---|---|---|
| 0.00001 | 105,49 | 95,6 | 101,46 | 98,53 | 104,1 | 96,64 | 102,98 | 97,01 | 100 | 92,97 | 99,17 | 107,85 |
| 0.0001 | 99,63 | 95,97 | 104,02 | 108,79 | 89,92 | 101,11 | 107,08 | 105,59 | 116,52 | 114,46 | 118,59 | 109,09 |
| 0.001 | 85,34 | 102,56 | 107,32 | 109,52 | 86,56 | 110,07 | 107,08 | 108,2 | 52,89 | 45,86 | 44,21 | 56,61 |
| 0.01 | 91,94 | 107,32 | 106,59 | 105,12 | 66,04 | 63,8 | 69,4 | 66,41 | 21,07 | 22,31 | 18,59 | 20,24 |
| 0.1 | 88,27 | 105,86 | 105,12 | 89,37 | 35,07 | 48,5 | 55,59 | 55,22 | 14,04 | 15,7 | 17,76 | 22,72 |
| 1 | 84,98 | 89,01 | 111,35 | 115,38 | 33,95 | 48,13 | 59,32 | 48,13 | 14,87 | 15,28 | 14,87 | 17,35 |
| 10 | 13,91 | 14,28 | 13,55 | 13,18 | 8,58 | 9,32 | 10,07 | 9,32 | 10,33 | 11,15 | 10,74 | 11,98 |
| 100 | 14,28 | 16,11 | 14,28 | 15,01 | 8,2 | 10,07 | 10,82 | 11,94 | 11,57 | 12,39 | 11,15 | 11,98 |

**PC9/DR**

| daco (μM) | DMSO | | | | CsA 1 μM | | | | CsA 10 μM | | | |
|---|---|---|---|---|---|---|---|---|---|---|---|---|
| 0.01 | 106 | 104 | 99 | 101 | 109 | 93 | 113 | 101 | 88 | 102 | 104 | 96 |
| 0.1 | 89 | 93 | 98 | 105 | 106 | 93 | 79 | 95 | 85 | 80 | 93 | 104 |
| 1 | 101 | 87 | 106 | 95 | 68 | 73 | 57 | 55 | 15 | 17 | 15 | 15 |
| 10 | 14,5 | 14,6 | 20,7 | 18,4 | 7,2 | 7,6 | 7,3 | 7,8 | 7,5 | 7,2 | 8,4 | 8,3 |
| 100 | 5,6 | 5,4 | 5,5 | 6,3 | 6,7 | 6,4 | 5,9 | 6,9 | 6 | 6,1 | 6,4 | 7,6 |

**PC9/OR**

| osi (μM) | DMSO | | | | CsA 10 μM | | | | CsA 20 μM | | | |
|---|---|---|---|---|---|---|---|---|---|---|---|---|
| 0.001 | 85,26 | 109,6 | 110,5 | 94,5 | 112,7 | 87,7 | 106,3 | 92,9 | 100,84 | 91,5 | 102,5 | 105 |
| 0.01 | 90 | 122,36 | 117,12 | 111,4 | 121,34 | 96,57 | 102,7 | 105,02 | 63,9 | 88,64 | 81,06 | 88,72 |
| 0.1 | 71,8 | 113 | 99,56 | 104,8 | 77,51 | 91,66 | 82,42 | 78,31 | 37,37 | 41,1 | 43,98 | 38,64 |
| 1 | 74,73 | 109,8 | 103,7 | 109,2 | 90,98 | 87,44 | 94,17 | 87,67 | 29,66 | 38,47 | 41,86 | 40 |
| 10 | 4,78 | 5,37 | 6,34 | 5,69 | 6,83 | 6,22 | 6,3 | 6,96 | 15,25 | 15,42 | 13,72 | 16,94 |
| 100 | 4,67 | 5 | 5,91 | 5,12 | 5,65 | 7,19 | 5,65 | 7,07 | 13,22 | 13,98 | 16,45 | 14,49 |

**PC9/GR**

| NT | CsA 1 μM | CsA 5 μM |
|---|---|---|
| 100,6034435 | 83,44128882 | 78,23728063 |
| 97,0824337 | 83,66273598 | 83,75131484 |
| 102,3085866 | 87,40519294 | 88,00310026 |
| 100,0055362 | 84,88069534 | 85,85506284 |

**PC9/DR**

| NT | CsA 1 μM | CsA 10 μM |
|---|---|---|
| 99,66842035 | 107,7147531 | 79,43764092 |
| 96,18462355 | 101,8082143 | 85,69786463 |
| 102,5155842 | 104,0541138 | 93,14293293 |
| 101,6313719 | 103,8065343 | 87,89601662 |

**PC9/OR**

| NT | CsA 10 μM | CsA 20 μM |
|---|---|---|
| 96,32949879 | 108,3910604 | 95,89066859 |
| 103,8146883 | 107,425634 | 89,6843557 |
| 95,7025985 | 104,2785945 | 90,54947811 |
| 104,1532144 | 102,6361157 | 86,52477823 |

# Figure 3 B

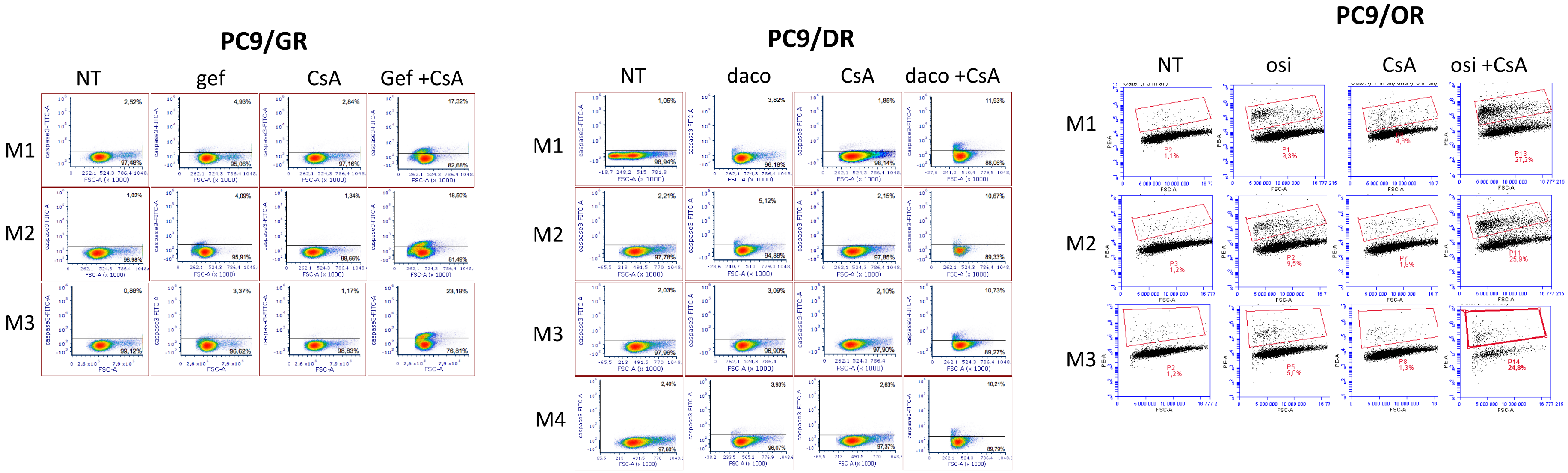


# Figure 3 D

Spheroid area (μM)

| NT | Osi | CsA | Osi + CsA |
|---|---|---|---|
| 672950 | 660365 | 576700 | 588320 |
| 708569 | 739127 | 729838 | 263953 |
| 727539 | 679295 | 678299 | 288255 |
| 599095 | 699606 | 625702 | 369199 |
