# Supplementary material for: PPP3CB overexpression mediates EGFR TKI resistance in lung tumors via calcineurin/MEK/ERK signaling
Source: Life Sci Alliance. 2024 Oct 1;7(12):e202402873. doi: 10.26508/lsa.202402873 (PMC11447527; doi:10.26508/lsa.202402873)

Figure 4 A

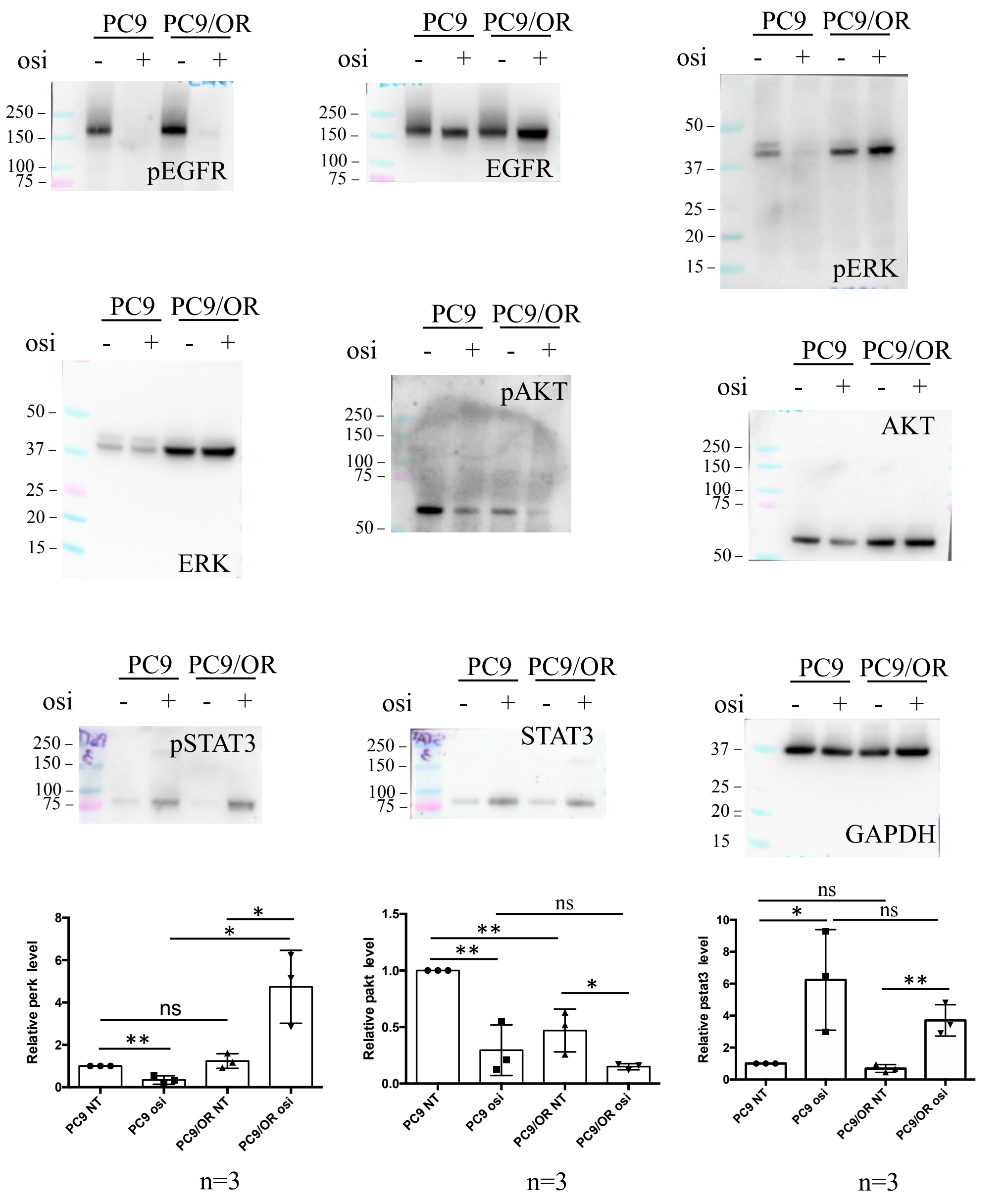

Figure 4 B

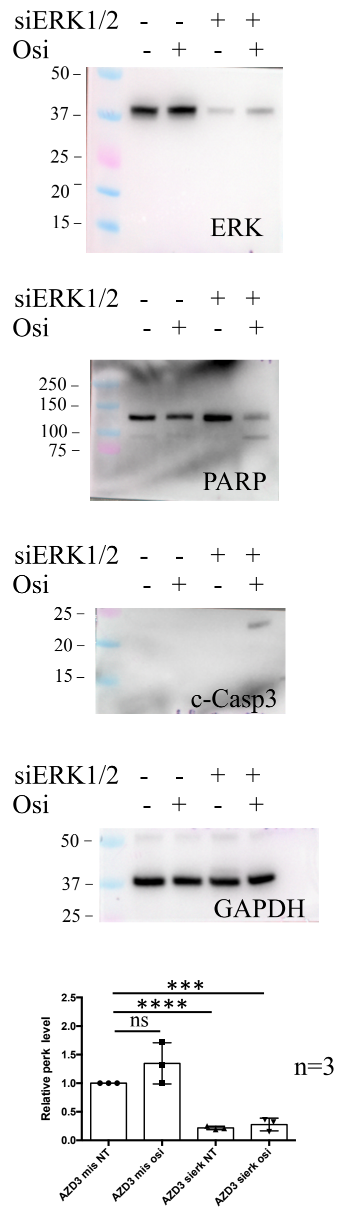

Figure 4 C

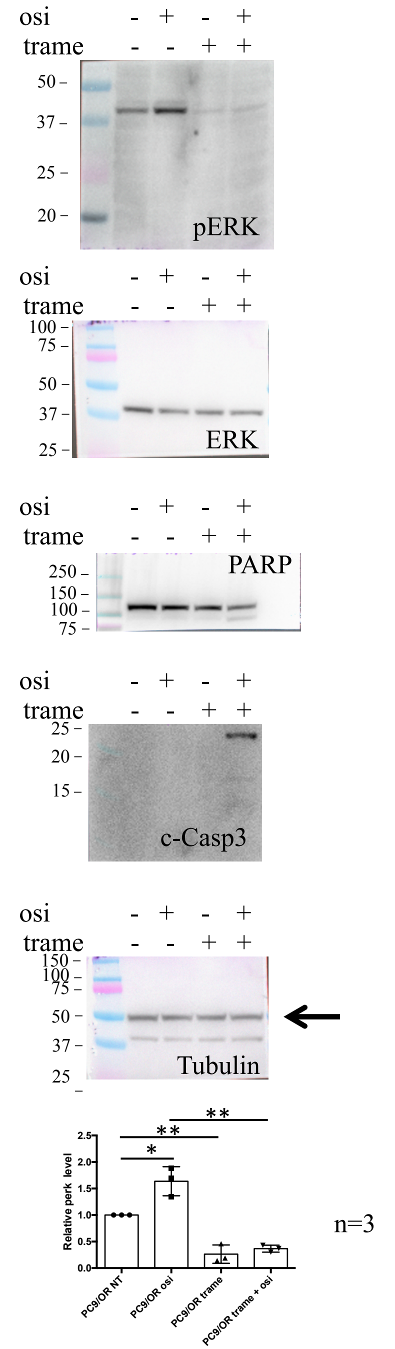

Figure 4 D

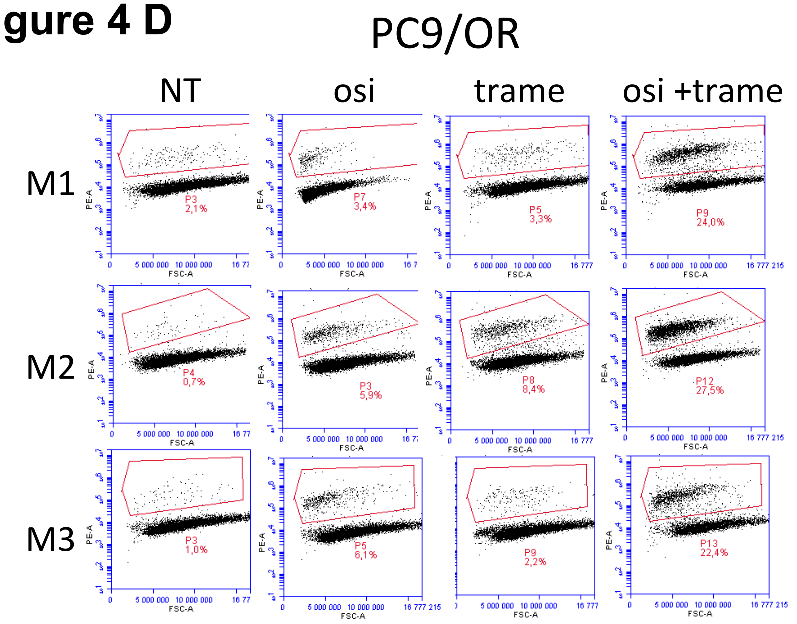

Figure 4 F

Spheroid area (μM)

NT	osi	trame	osi + trame
161906	163200	124687	118797
157340	163632	126397	62810
161026	158810	154944	78465
155992	151755	107052	111545

Figure 4 G

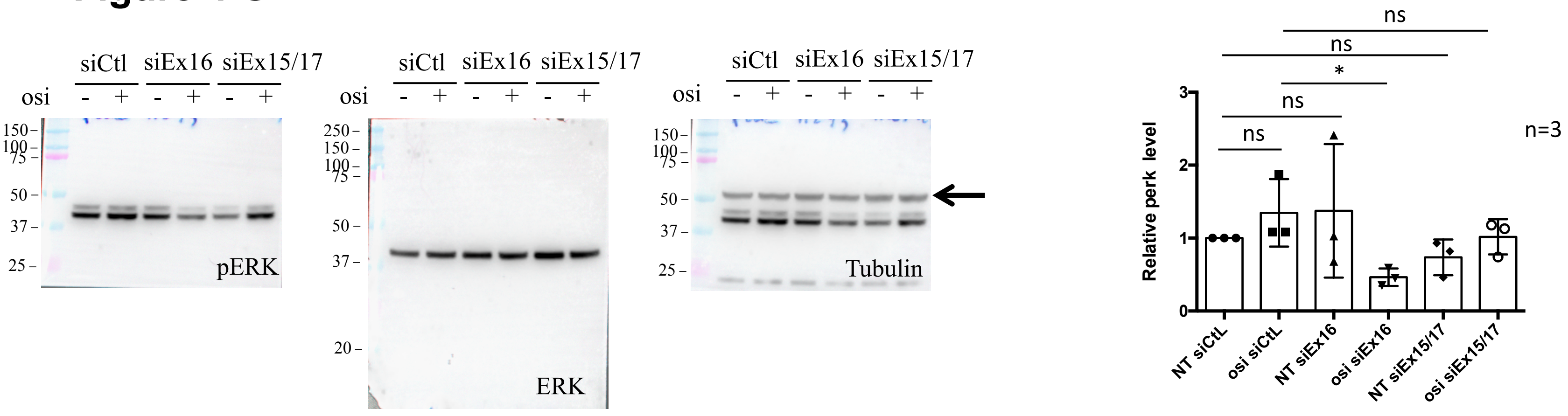

Figure 4 H

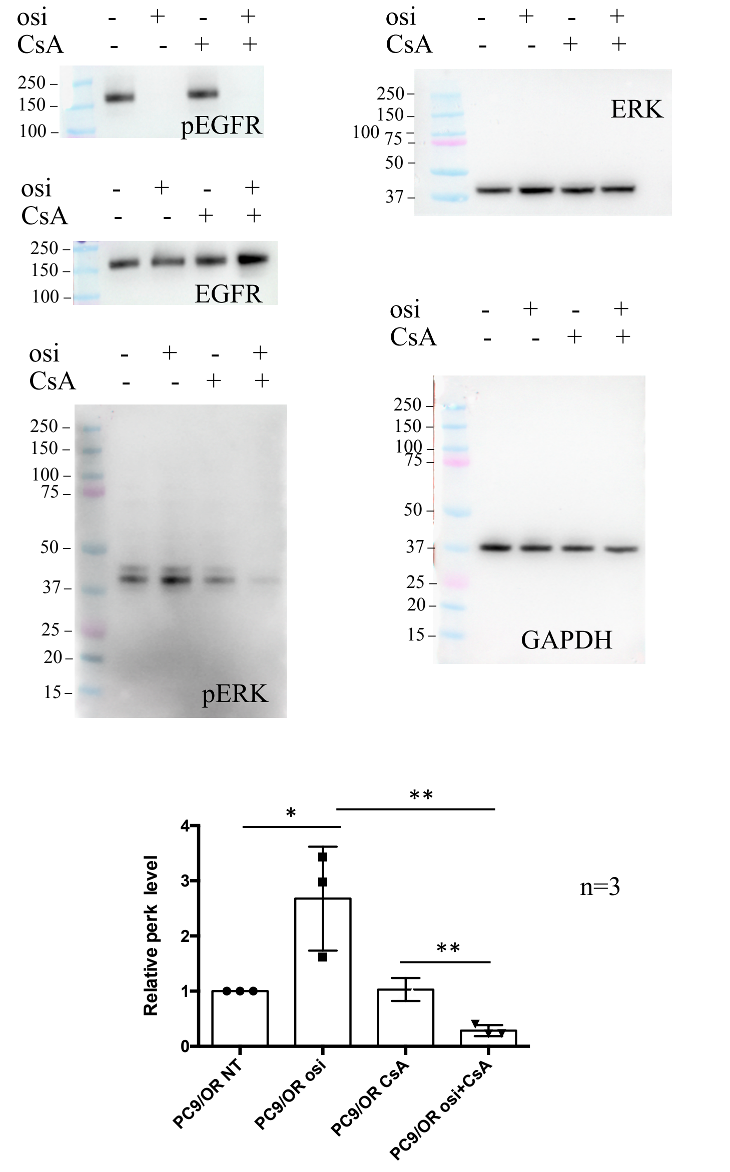

Figure 4 I

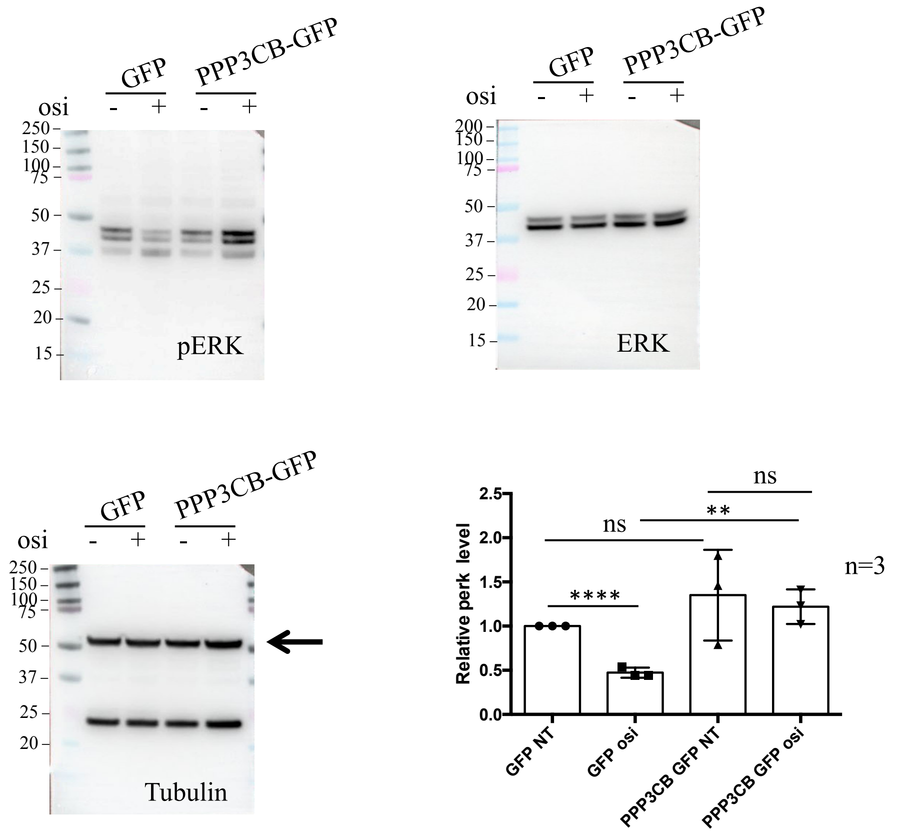

Figure 4 J

Relative calcium level

NT	osi 0.01 μM	osi 0.1 μM	osi 1 μM	osi 10 μM
1	1.148	1.73	6.3	5.34
1	1.71	4.4	3.1	4.58
1	2.16	3.29	2.7	5.35
1	1.44	3.44	4.2	5,2

Figure 4 L

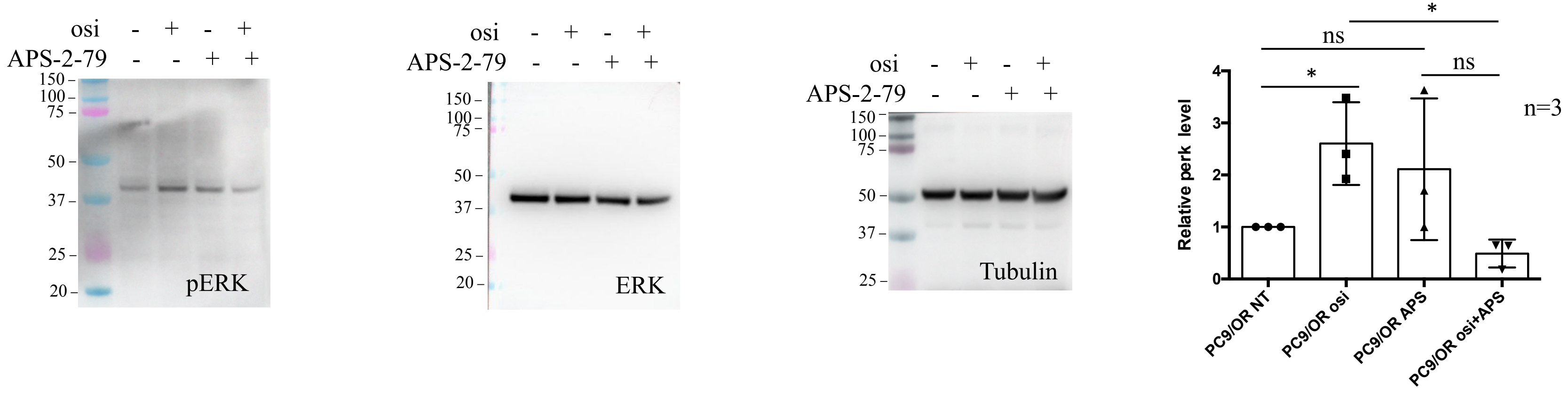

Supplement: Supplementary file 6 [file LSA-2024-02873_SdataF4.pdf]
