# Supplementary material for: PPP3CB overexpression mediates EGFR TKI resistance in lung tumors via calcineurin/MEK/ERK signaling
Source: Life Sci Alliance. 2024 Oct 1;7(12):e202402873. doi: 10.26508/lsa.202402873 (PMC11447527; doi:10.26508/lsa.202402873)
Supplement: Supplementary file 7 [file LSA-2024-02873_SdataFS2.pptx]

## Slide 1
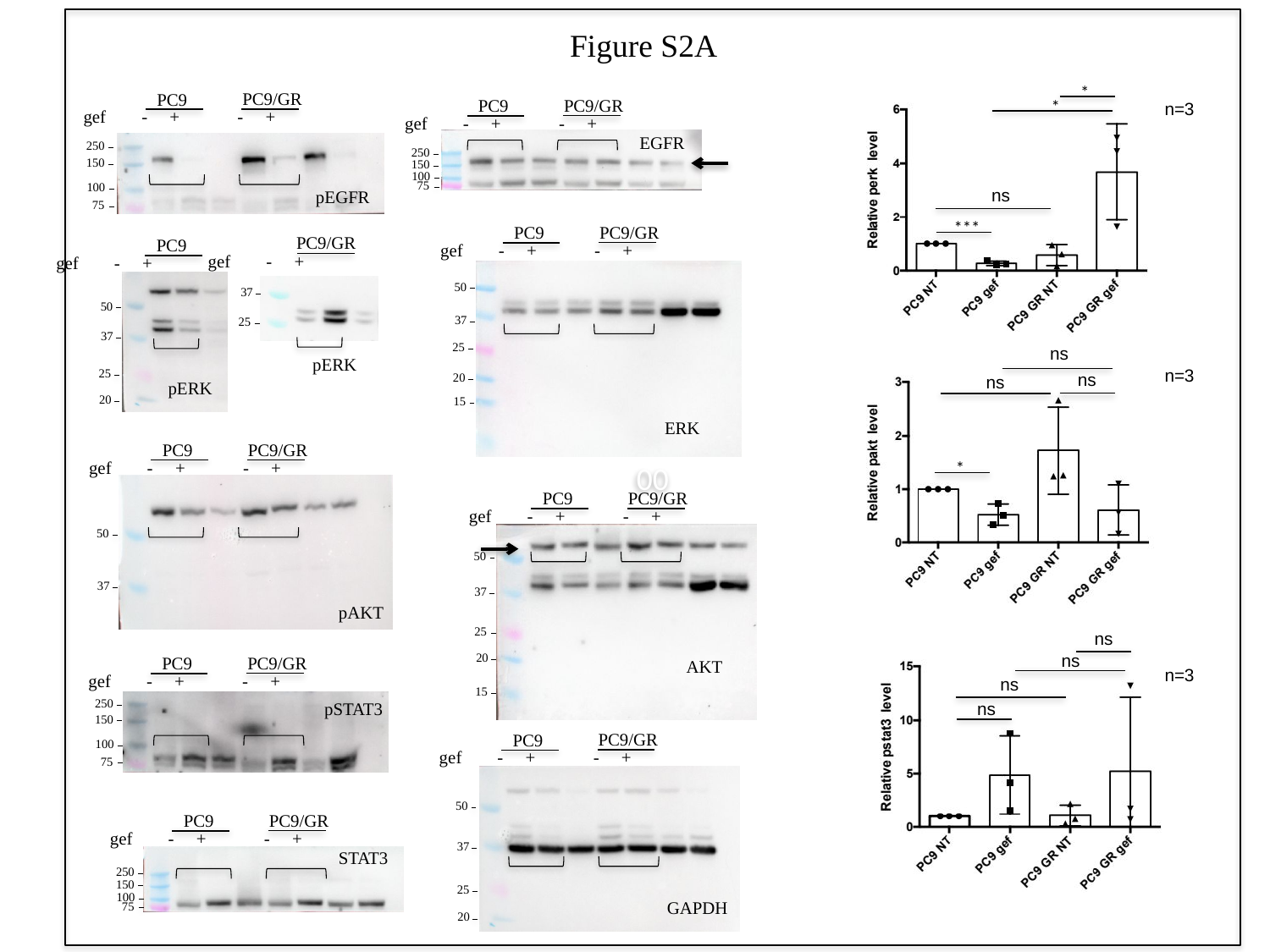

00
Figure S2A
*
*
ns
***
PC9/GR
PC9
PC9/GR
PC9
n=3
gef - + - +
gef - + - +
EGFR
250
250
150
150
100
75
100
pEGFR
75
PC9/GR
PC9
gef - + - +
ERK
PC9/GR
PC9
gef - +
gef - +
50
37
50
37
25
37
25
ns
ns
ns
*
pERK
n=3
25
20
pERK
20
15
PC9/GR
PC9
gef - + - +
PC9/GR
PC9
gef - + - +
AKT
50
50
37
37
pAKT
25
ns
ns
ns
ns
20
PC9/GR
PC9
gef - + - +
pSTAT3
n=3
15
250
150
PC9/GR
PC9
100
gef - + - +
75
50
PC9/GR
PC9
gef - + - +
STAT3
37
250
150
25
100
GAPDH
75
20

## Slide 2
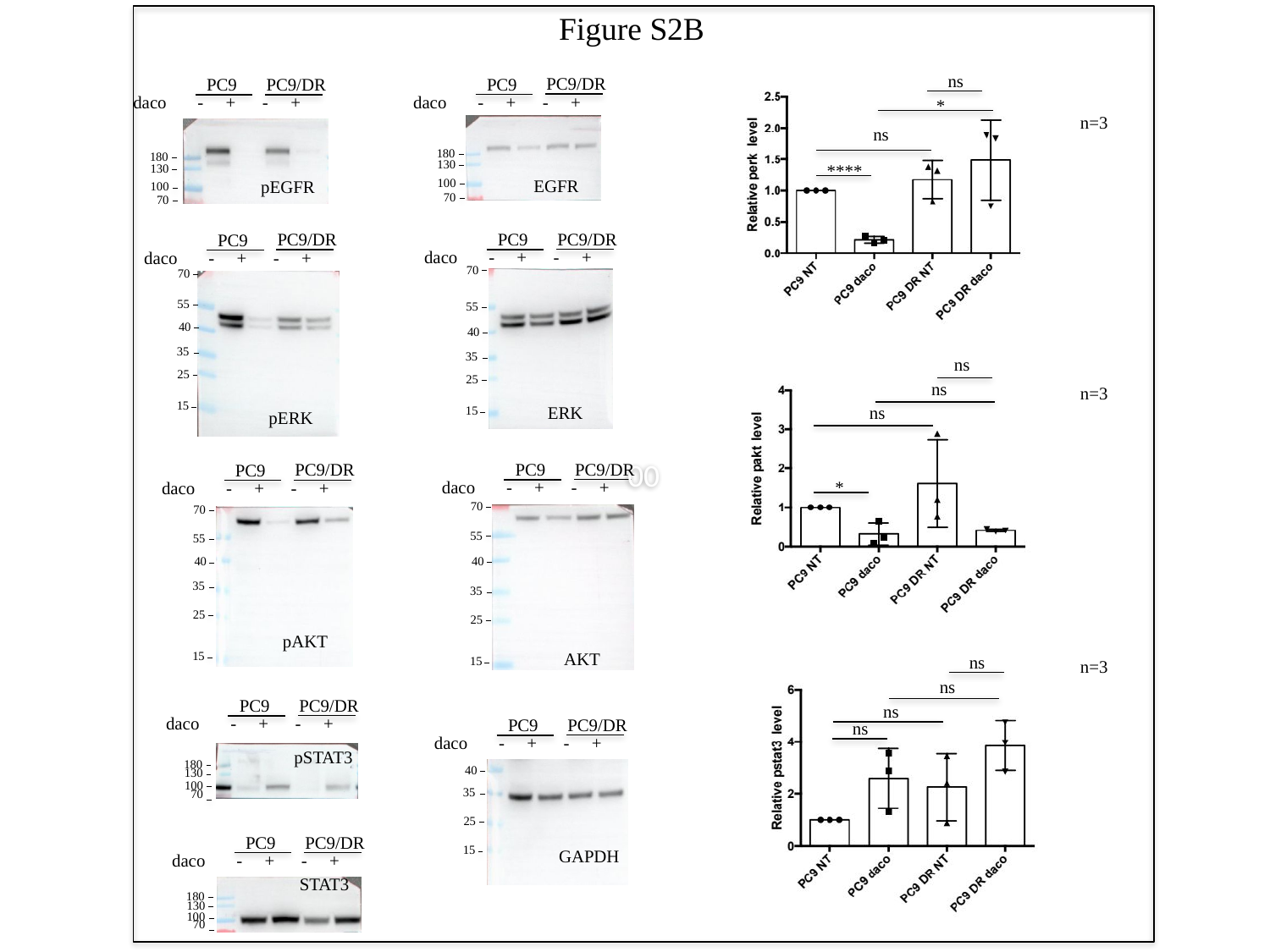

Figure S2B
00
ns
*
ns
****
PC9/DR
PC9
PC9/DR
PC9
daco - + - +
daco - + - +
n=3
180
180
130
130
100
EGFR
pEGFR
100
70
70
PC9/DR
PC9
PC9/DR
PC9
daco - + - +
daco - + - +
70
70
55
55
40
40
35
35
ns
ns
ns
*
25
25
n=3
15
ERK
15
pERK
PC9/DR
PC9
PC9/DR
PC9
daco - + - +
daco - + - +
70
70
55
55
40
40
35
35
25
25
pAKT
AKT
15
ns
ns
ns
ns
15
n=3
PC9/DR
PC9
daco - + - +
PC9/DR
PC9
daco - + - +
pSTAT3
180
40
130
100
35
70
25
PC9/DR
PC9
15
GAPDH
daco - + - +
STAT3
180
130
100
70
