# Supplementary material for: PPP3CB overexpression mediates EGFR TKI resistance in lung tumors via calcineurin/MEK/ERK signaling
Source: Life Sci Alliance. 2024 Oct 1;7(12):e202402873. doi: 10.26508/lsa.202402873 (PMC11447527; doi:10.26508/lsa.202402873)
Supplement: Supplementary file 8 [file LSA-2024-02873_SdataFS3.pptx]

## Slide 1
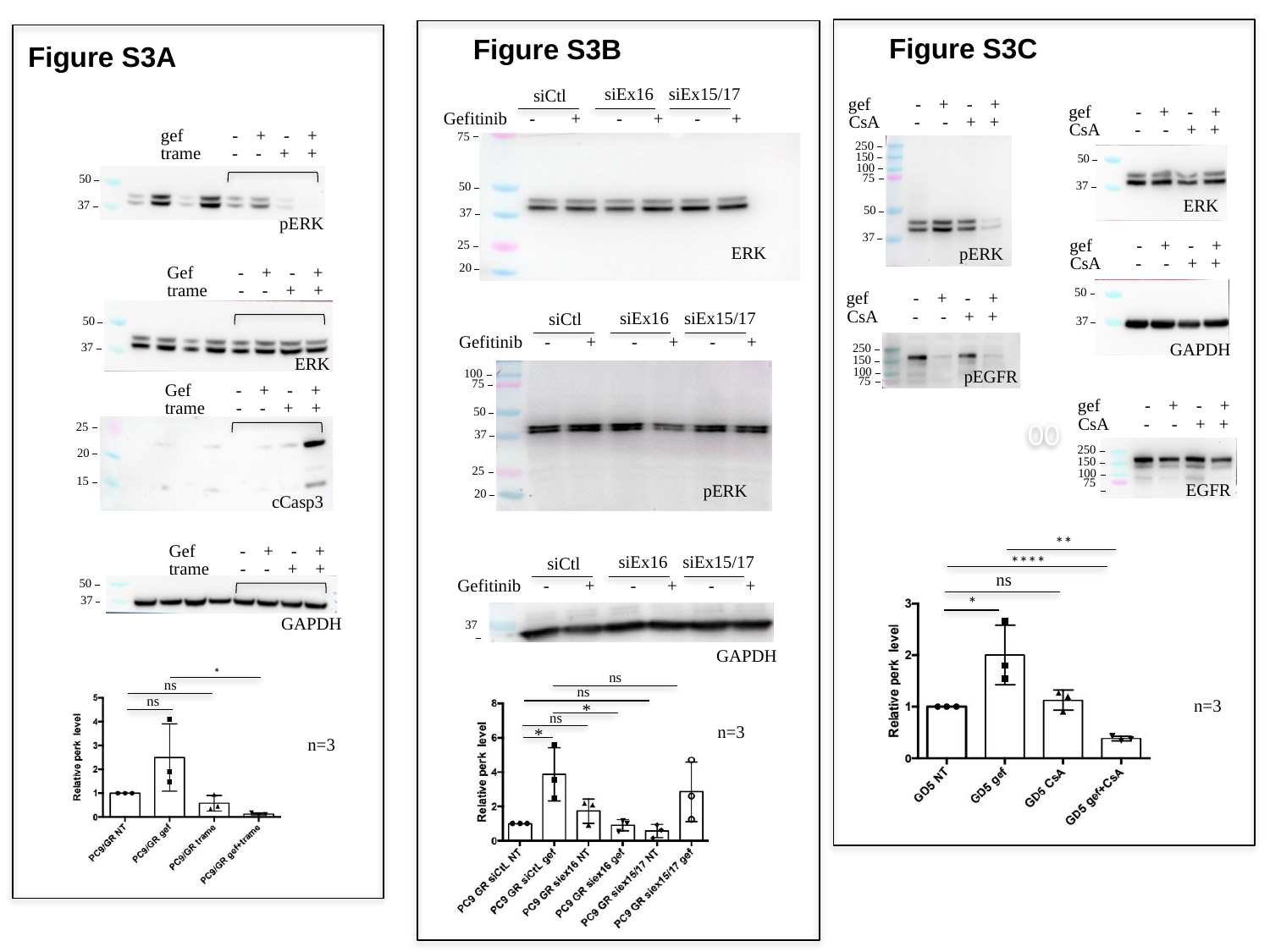

00
Figure S3C
Figure S3B
Figure S3A
siEx16
siEx15/17
siCtl
gef - + - +
gef - + - +
Gefitinib - + - + - +
CsA - - + +
CsA - - + +
gef - + - +
75
250
trame - - + +
150
50
100
75
50
37
50
ERK
37
50
37
pERK
37
gef - + - +
25
ERK
pERK
CsA - - + +
20
Gef - + - +
trame - - + +
ERK
50
gef - + - +
CsA - - + +
siEx16
siEx15/17
siCtl
50
37
Gefitinib - + - + - +
GAPDH
37
250
150
100
pEGFR
100
75
75
Gef - + - +
trame - - + +
cCasp3
gef - + - +
50
CsA - - + +
25
37
250
20
150
25
100
15
75
EGFR
pERK
20
**
Gef - + - +
siEx16
siEx15/17
****
siCtl
trame - - + +
ns
Gefitinib - + - + - +
50
*
37
GAPDH
37
GAPDH
*
ns
ns
ns
ns
n=3
*
ns
n=3
*
n=3

## Slide 2
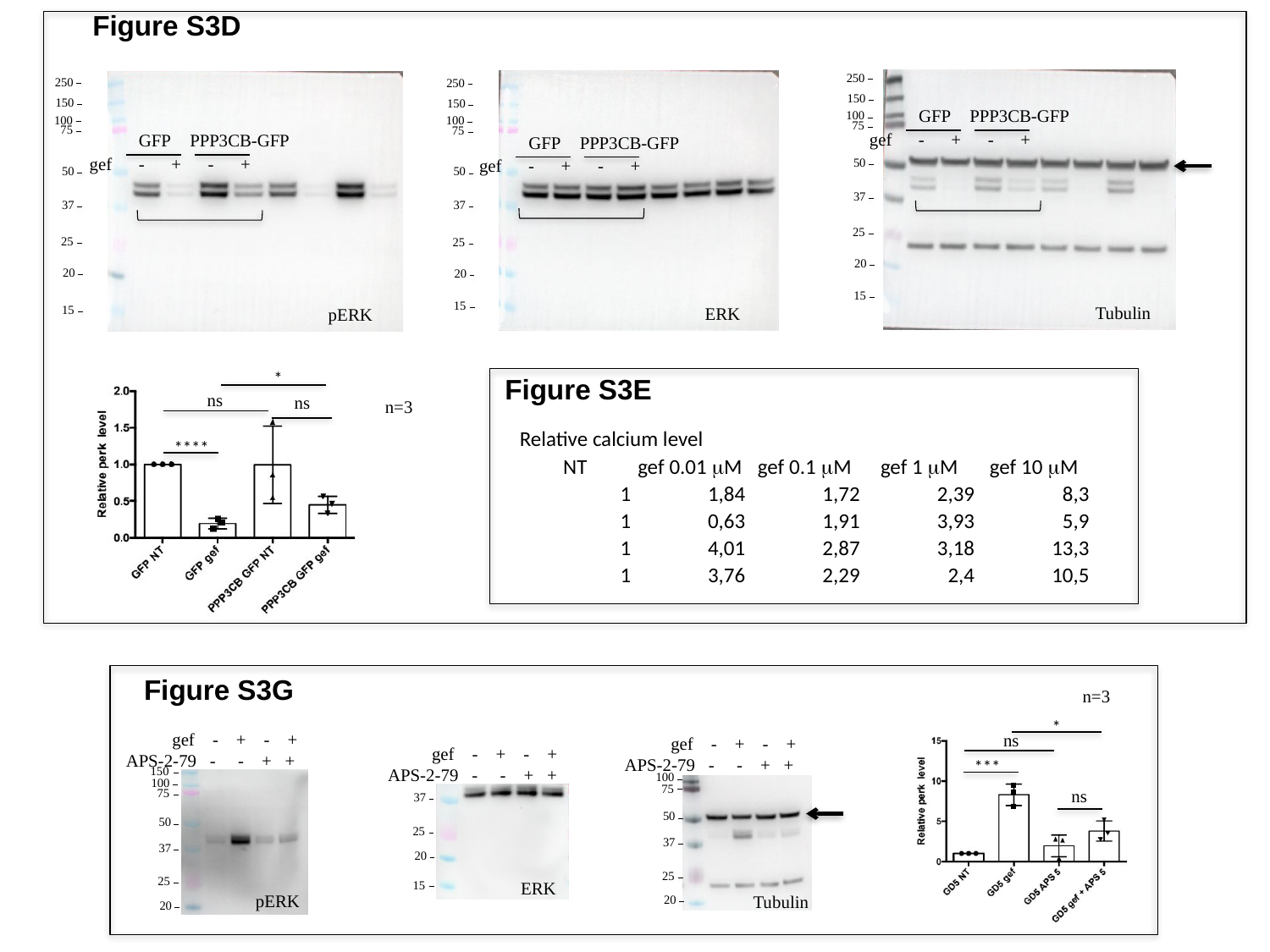

Figure S3D
250
150
GFP
PPP3CB-GFP
100
75
gef - + - +
50
37
25
20
15
Tubulin
250
150
100
75
GFP
PPP3CB-GFP
gef - + - +
50
37
25
20
15
pERK
250
150
100
75
GFP
PPP3CB-GFP
gef - + - +
50
37
25
20
15
ERK
*
Figure S3E
ns
ns
n=3
| Relative calcium level | | | | |
| --- | --- | --- | --- | --- |
| NT | gef 0.01 mM | gef 0.1 mM | gef 1 mM | gef 10 mM |
| 1 | 1,84 | 1,72 | 2,39 | 8,3 |
| 1 | 0,63 | 1,91 | 3,93 | 5,9 |
| 1 | 4,01 | 2,87 | 3,18 | 13,3 |
| 1 | 3,76 | 2,29 | 2,4 | 10,5 |
****
Figure S3G
n=3
*
gef - + - +
APS-2-79 - - + +
150
100
75
50
37
25
pERK
20
ns
gef - + - +
APS-2-79 - - + +
100
75
50
37
25
Tubulin
20
gef - + - +
APS-2-79 - - + +
37
25
20
15
ERK
***
ns

## Slide 3
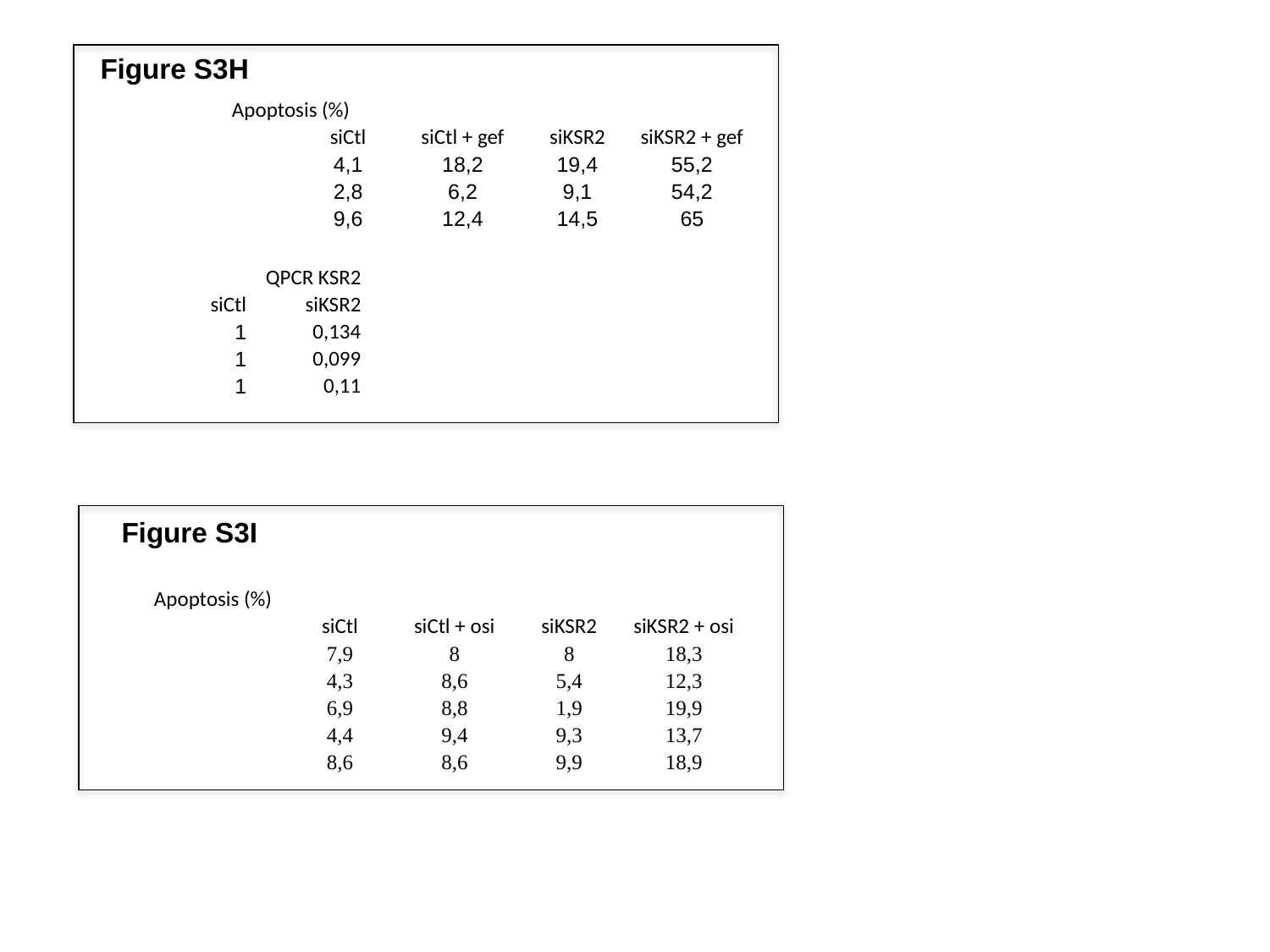

Figure S3H
| Apoptosis (%) | | | | |
| --- | --- | --- | --- | --- |
| | siCtl | siCtl + gef | siKSR2 | siKSR2 + gef |
| | 4,1 | 18,2 | 19,4 | 55,2 |
| | 2,8 | 6,2 | 9,1 | 54,2 |
| | 9,6 | 12,4 | 14,5 | 65 |
| QPCR KSR2 | |
| --- | --- |
| siCtl | siKSR2 |
| 1 | 0,134 |
| 1 | 0,099 |
| 1 | 0,11 |
Figure S3I
| Apoptosis (%) | | | | |
| --- | --- | --- | --- | --- |
| | siCtl | siCtl + osi | siKSR2 | siKSR2 + osi |
| | 7,9 | 8 | 8 | 18,3 |
| | 4,3 | 8,6 | 5,4 | 12,3 |
| | 6,9 | 8,8 | 1,9 | 19,9 |
| | 4,4 | 9,4 | 9,3 | 13,7 |
| | 8,6 | 8,6 | 9,9 | 18,9 |
