# Supplementary material for: PPP3CB overexpression mediates EGFR TKI resistance in lung tumors via calcineurin/MEK/ERK signaling
Source: Life Sci Alliance. 2024 Oct 1;7(12):e202402873. doi: 10.26508/lsa.202402873 (PMC11447527; doi:10.26508/lsa.202402873)
Supplement: Supplementary file 9 [file LSA-2024-02873_SdataF5.pdf]

Figure 5A

% of living cells

osi (μM)	Vehicle				CsA				trame				CsA + trame			
0.00001	84,2134	108,173	100,4776	107,136	95,28895	103,706	106,3442	94,66081	85,60259	99,54105	111,4397	103,4166	94,54253	103,9593	99,30444	102,1937
0.0001	84,86833	99,33142	123,2365	122,1449	110,9297	106,4699	108,4171	101,8216	93,01377	89,41016	104,8445	102,1928	121,1343	111,0754	95,66613	106,046
0.001	84,81375	106,2628	129,4583	129,24	126,5704	140,9548	136,2437	94,72362	99,47306	92,26585	96,27741	105,8644	124,8261	123,649	97,85982	92,40235
0.01	82,08487	99,386	157,6204	158,1116	173,4296	164,5729	163,5678	157,098	190,3111	167,3976	172,2931	186,9794	48,42162	52,70198	44,03425	45,10434
0.1	124,2734	135,8985	141,0834	149,8704	163,5678	151,9472	137,9397	132,098	163,1141	143,1243	140,6765	156,5188	46,81648	43,17817	40,44944	35,36651
1	123,8914	148,9971	142,8844	147,6873	154,1457	147,9271	151,1935	133,794	161,6182	153,3231	151,0114	135,3051	52,16694	51,41787	36,65062	39,48636
10	132,7534	130,8673	134,9707	131,2594	160,4899	148,4925	147,6759	121,2312	165,2898	148,5637	148,2917	150,2635	36,97164	37,6137	31,13965	30,5511
100	123,8914	133,6062	137,9179	130,0587	167,3367	91,83417	115,5151	100,8166	146,7959	142,2404	146,7959	150,1955	23,59551	21,1343	25,46816	23,70252

	DMSO	trame	CsA	trame+CsA
IC50 M1	5,7	6,03	3,78	0,0045
IC50 M2	9,8	9,01	6,06	0,0087
IC50 M3	9,5	6,07	1,81	0,002

Figure 5D

Spheroid area (μm)

NT	osi	CsA	trame	osi + CsA	osi + trame	osi + CsA + trame
288118	292029	279540	309661	240301	311574	173223
276517	286443	294894	284001	229159	307846	93848
291366	289942	278515	319167	228387	300033	99494
274046	272162	280232	334680	247442	311407	195311

Figure 5E

Cell viability (%)

NT	osi	CsA	trame	osi + CsA	osi + trame	osi + CsA + trame
857666	1107197	1241263	1032857	1052287	1186710	416215
876445	1022000	1446191	1284260	1105843	1152946	49462
783996	1200035	1458840	765153	936798	1205068	13753
813622	1467468	1481631	1085255	1100940	1182863	376157
