# Supplementary material for: PPP3CB overexpression mediates EGFR TKI resistance in lung tumors via calcineurin/MEK/ERK signaling
Source: Life Sci Alliance. 2024 Oct 1;7(12):e202402873. doi: 10.26508/lsa.202402873 (PMC11447527; doi:10.26508/lsa.202402873)
Supplement: Supplementary file 10 [file LSA-2024-02873_SdataF6.pdf]

Figure 6A

VOLUME (mm³)

	Gpe 1: Control								
	1	4	8	11	15	18	22	25	29
44	126	225	253	320	446	625	860	1094	1268
12	69	108	184	208	239	288	600	600	600
16	69	137	196	256	361	425	726	936	1008
2	75	117	158	208	320	336	379	726	1008
10	75	75	148	225	405	425	696	726	1470
18	91	169	233	361	397	564	1008	1080	1764
49	108	148	256	304	405	405	550	900	1268
5	63	108	108	196	196	288	500	726	1094
59	69	137	225	272	405	525	600	787	1372
40	75	126	208	253	253	405	600	787	1094
Med	*75*	132	202	255	379	415	600	787	1181
Mean	*82*	*135*	*197*	*260*	*343*	*429*	*652*	*836*	*1194*

	Gpe 2: Ciclosporine A 25mg/kg								
	1	4	8	11	15	18	22	25	29
3	63	108	117	144	239	343	405	550	466
22	69	137	148	158	208	309	446	446	662
27	69	91	135	144	208	253	343	425	486
13	75	148	184	253	288	320	525	606	726
19	75	117	184	196	288	288	405	500	550
24	91	117	196	272	385	385	451	666	900
1	75	113	184	208	325	325	385	425	550
75	91	144	196	288	288	446	466	650	1080
Med	*75*	117	184	196	288	323	425	525	606
Mean	*79*	*121*	*163*	*199*	*268*	*334*	*428*	*533*	*677*

	Gpe 3: Trametinib 0,1mg/kg								
	1	4	8	11	15	18	22	25	29
6	63	81	135	144	190	281	451	542	860
32	69	108	184	256	385	525	936	1470	1913
37	69	108	184	184	288	304	425	606	756
20	75	106	144	211	352	466	772	968	1862
26	75	108	169	196	196	320	446	446	717
43	91	148	196	256	256	343	550	794	1008
7	98	144	221	288	405	425	726	726	1094
28	75	88	144	221	281	446	446	486	650
83	117	117	126	169	196	361	361	486	717
Med	*75*	*108*	*169*	*211*	*281*	*361*	*451*	*606*	*860*
Mean	*81*	*112*	*167*	*214*	*283*	*386*	*568*	*725*	*1064*

	Gpe 4: Osimertinib 5mg/kg								
	1	4	8	11	15	18	22	25	29
23	63	148	135	172	225	272	425	446	600
46	69	69	148	148	256	256	325	361	451
51	69	75	75	126	196	184	196	256	325
29	75	91	91	83	69	91	126	75	106
31	75	126	169	239	320	343	451	500	550
58	91	108	126	169	148	196	320	385	405
66	98	106	113	158	196	253	343	385	446
87	108	126	91	117	108	172	144	184	196
15	75	108	91	126	126	211	256	272	272
Med	*75*	108	113	148	196	211	320	361	405
Mean	*80*	*106*	*115*	*149*	*183*	*220*	*287*	*318*	*372*

Figure 6A

	Gpe 5: Ciclosporine A 25mg/kg + Osimertinib 5mg/kg								
	1	4	8	11	15	18	22	25	29
39	63	63	51	51	63	63	91	75	69
55	69	69	69	69	94	75	126	144	196
57	69	117	117	184	288	196	256	288	325
33	75	91	91	63	108	75	63	108	117
38	75	75	83	63	32	63	75	14	14
69	91	144	196	196	245	288	405	320	446
9	117	106	196	153	245	304	425	446	600
4	81	98	66	126	75	126	75	126	196
62	75	88	69	63	63	63	63	75	75
71	98	91	69	91	117	108	148	196	288
Med	*75*	91	76	80	101	92	108	135	196
Mean	*81*	*94*	*101*	*106*	*133*	*136*	*173*	*179*	*232*

	Gpe 6: Osimertinib 5mg/kg + Trametinib 0,1mg/kg								
	1	4	8	11	15	18	22	25	29
73	63	69	75	75	126	126	196	126	196
64	69	75	69	69	69	69	126	75	81
65	69	63	63	63	63	91	117	108	75
56	75	91	98	88	148	158	225	196	196
68	75	91	91	75	91	108	172	117	108
21	108	137	126	288	325	253	253	253	288
34	117	117	98	108	117	117	196	184	225
81	69	117	137	184	208	295	352	600	600
11	75	81	75	126	88	126	108	117	91
30	91	63	91	75	81	40	75	69	75
Med	*75*	86	91	81	104	122	184	122	152
Mean	*81*	*90*	*92*	*115*	*132*	*138*	*182*	*184*	*194*

	Gpe 7: Ciclosporine A 25mg/kg + Trametinib 0,1mg/kg								
	1	4	8	11	15	18	22	25	29
77	63	88	126	196	288	288	405	446	486
70	69	126	211	272	451	525	794	936	1352
72	69	91	172	184	365	365	500	606	799
85	75	148	288	361	550	550	717	787	1080
41	69	126	148	196	267	496	575	936	726
25	108	184	253	325	496	550	600	787	817
53	117	225	365	365	525	696	1055	1183	1470
60	69	126	184	253	288	451	385	500	726
14	75	106	148	169	239	288	405	496	600
Med	*69*	126	184	253	365	496	575	787	799
Mean	*79*	*135*	*210*	*258*	*385*	*468*	*604*	*742*	*895*

	Gpe 8: Ciclosporine A AM 25mg/kg + Osimertinib AM 5mg/kg + Trametinib PM 0,1								
	1	4	8	11	15	18	22	25	29
78	63	83	4	0	4	0	63	32	75
76	69	75	32	1	4	4	14	1	4
86	75	75	46	46	69	69	63	126	172
45	83	63	1	0	1	0	0	4	32
36	75	91	36	4	18	14	108	63	108
61	117	126	63	32	32	32	63	14	40
63	169	144	69	32	25	18	51	32	32
54	63	91	32	18	0	14	0	4	63
17	63	75	69	75	63	91	75	63	63
Med	*72*	79	34	11	18	14	63	32	63
Mean	*84*	*87*	*35*	*21*	*24*	*27*	*48*	*37*	*65*
