# Supplementary figures and images for: PPP3CB overexpression mediates EGFR TKI resistance in lung tumors via calcineurin/MEK/ERK signaling
Source: Life Sci Alliance. 2024 Oct 1;7(12):e202402873. doi: 10.26508/lsa.202402873 (PMC11447527; doi:10.26508/lsa.202402873)

## Slide 1
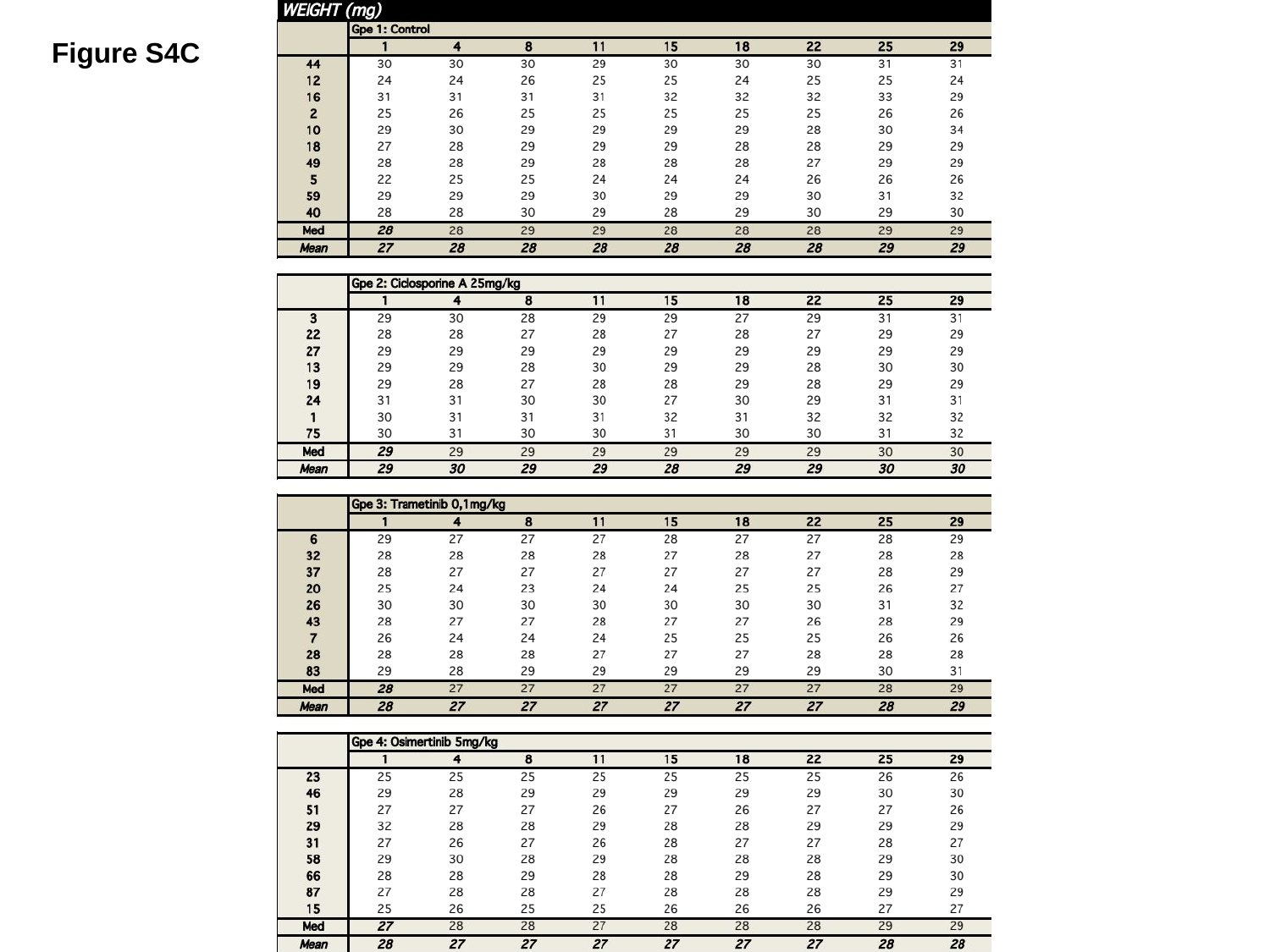

Figure S4C

## Slide 2
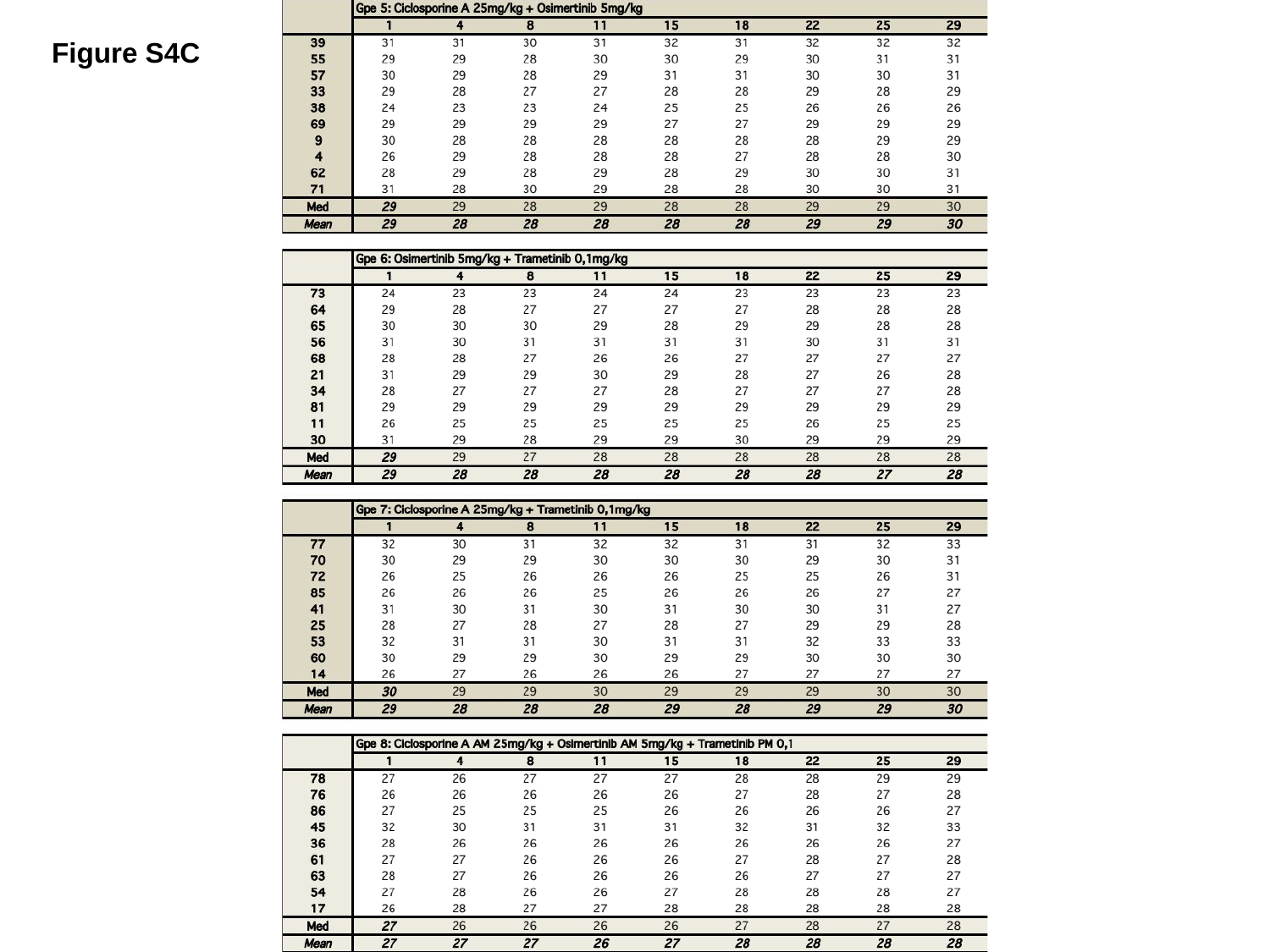

Figure S4C

Supplement: Supplementary file 11 [file LSA-2024-02873_SdataFS4.pptx]

## Slide 1
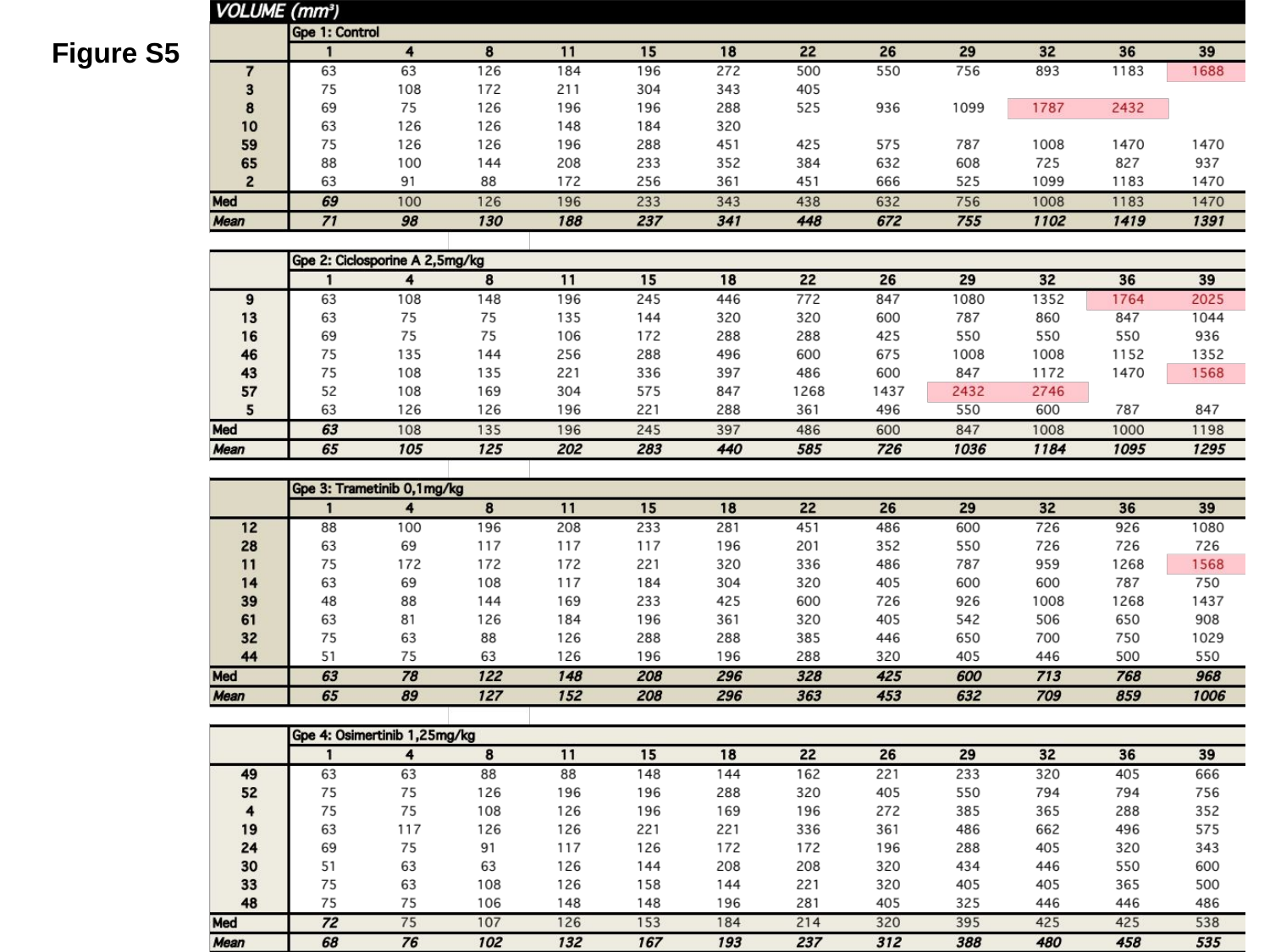

Figure S5

## Slide 2
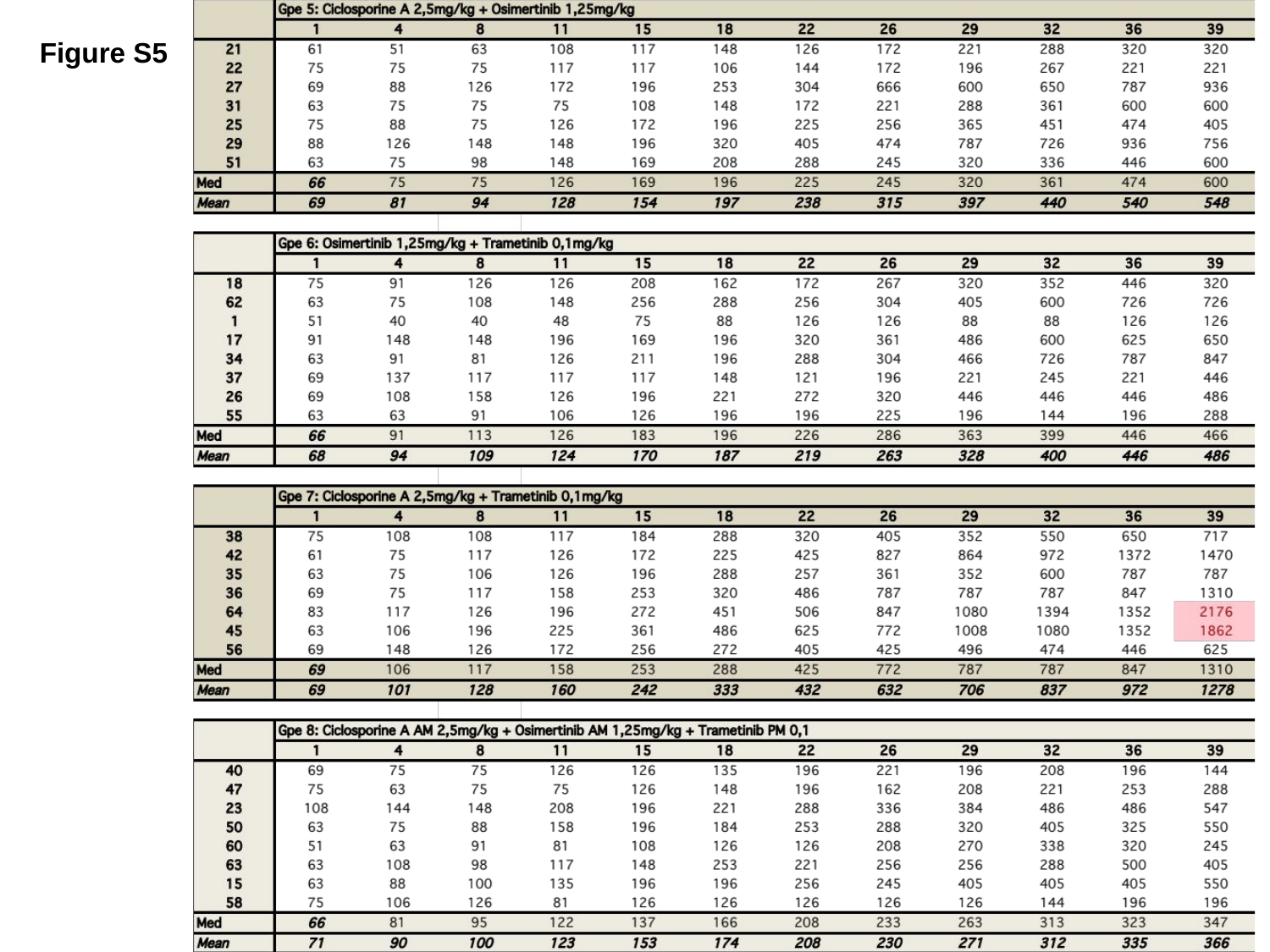

Figure S5

Supplement: Supplementary file 12 [file LSA-2024-02873_SdataFS5.pptx]

## Slide 1
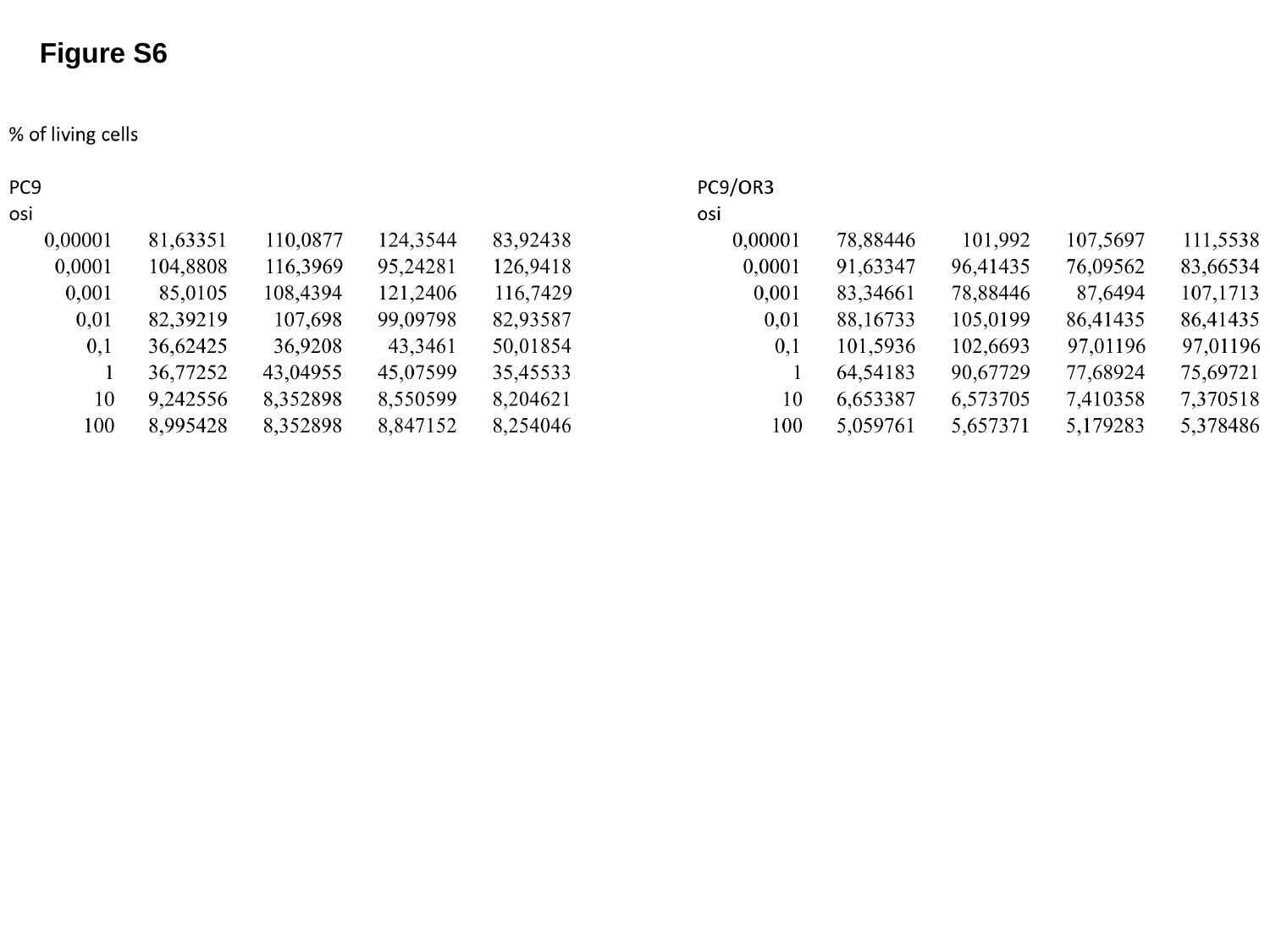

Figure S6

Supplement: Supplementary file 13 [file LSA-2024-02873_SdataFS6.pptx]
